# Experimental Investigations on Interface between Ordinary and Lightweight Aggregate Concretes Cast at Different Times

**DOI:** 10.3390/ma14071664

**Published:** 2021-03-28

**Authors:** Michał Gołdyn, Tadeusz Urban

**Affiliations:** Department of Concrete Structures, Lodz University of Technology, Politechniki 6, 90-924 Łódź, Poland; tadeusz.urban@p.lodz.pl

**Keywords:** interface, push-off test, shear behaviour, concrete cast at different time, lightweight aggregate concrete, post-installed reinforcement

## Abstract

Experimental investigations on 12 push-off specimens with dimensions of 600 × 300 × 180 mm (200 × 180 mm shear plane) were presented. Models reflected the connection between ordinary concrete (NWC) substrate and lightweight aggregate concrete (LWAC) overlay. The main purpose of the study was to investigate behaviour of the interface between concretes cast at different times. Two different interface conditions were considered: Smooth and rough (obtained by graining). In the selected elements, additional reinforcement consisting of one ∅8 bar was injected. The elements were tested under load control. The failure of the specimens without interface reinforcement was violent and resulted from breaking of the adhesive bond. Specimens with shear reinforcement failed in a ductile manner, however, due to the low reinforcement area, the residual load capacity was much lower than the load recorded just before cracking. It was found that mechanical roughening of the surface can lead to degradation of the concrete structure. As a result, the load-carrying capacities of elements with smooth interface proved to be higher than the ultimate loads of elements with deliberately roughened contacts. Comparative analysis showed that the existing design procedures ACI 318-19, Eurocode 2, Model Code 2010, and AASHTO can lead to safe but conservative estimation of the actual resistance of the concrete interface.

## 1. Introduction

**Figure 1:**
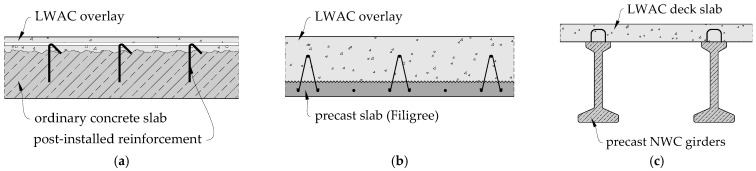
Examples of the use of lightweight aggregate concrete as structural overlay: (**a**) flat slab strengthened with concrete overlay; (**b**) composite slab with Filigree precast elements; (**c**) bridge decks.

During reconstruction of the existing buildings, it may be necessary to increase the load-bearing capacity of the structural elements [1,2]. In many cases, such a need may also result from construction errors, when the amount or location of the reinforcement differs from the assumptions made in the design documentation. This situation very often occurs in the flat slabs when the top (main) reinforcement is moved downwards. In some cases, the load capacity deficit resulting from the reduction of effective depth is so significant that the only rational way to strengthen the support zones relies on introducing additional longitudinal reinforcement located in the concrete overlay. However, the new layer of concrete will constitute an additional load, therefore it may be a rational solution to replace normal weight concrete (NWC) with lightweight aggregate concrete (LWAC) (see Figure 1a). Lightweight aggregate concrete can also be an alternative to ordinary concrete used as topping concrete in composite floors with Filigree or TT precast elements (Figure 1b). Increasingly, LWAC is also used in bridge structures [3], where the main load-bearing structure consists of precast NWC girders, while the road slab is made of lightweight aggregate concrete on site, thus creating a composite structure (see Figure 1c).

Depending on the density class (according to EN 1992-1-1 [4]), lightweight, structural aggregate concretes (density classes 1.6–2.0) are characterized by a specific gravity, lower by about 15–30% compared to ordinary concrete. Significant progress in concrete technology made it possible to obtain lightweight aggregate concretes with compressive strength equal to ordinary ones. However, a lower increase in tensile strength, accompanying the increase in compressive strength, is observed in LWAC compared to NWC. This feature may be important in the composite structures when the tensile strength plays a key role, determining the cracking load of the interface. This will be of particular importance in the case of non-reinforced interfaces where the load capacity depends on the adhesive forces. Previous experimental studies demonstrated that in the reinforced interfaces, the adhesive forces act only at very low slip. After cracking, the bearing capacity depends on the dowel action and the aggregate interlock effect. In case of LWAC, the second of the mechanisms mentioned can be limited. The aggregate is the weakest component of the composite and therefore is susceptible to cracking and crushing. The question of the load carrying capacity of the contact between concretes cast at different times seems justified when LWAC is used as the topping.

### 1.1. Experimental Investigations

The load carrying capacity of the interfaces between concretes cast at different times has been the subject of experimental research since the 1960s. The first studies in this field include the works by Kriz and Raths [5], Birkenland and Birkenland [6] (1966—shear-friction theory), Mast [7], Hofbeck et al. [8], and Mattock and Hawkins [9] (1972—modified shear-friction theory). Research on the connections of concretes cast at different times are still ongoing. The main variable parameters considered in the previous studies were listed in Table 1.

The shear resistance of the interface is clearly related to the compressive strength of concrete, which translates into both the size of the adhesive forces and the residual load capacity in the post-peak phase (after cracking). At high shear reinforcement ratios, concrete strength determines the strength of the diagonal struts and becomes one of the parameters governing of the residual load-carrying capacity of the connection [8,15].

The introduction of transverse reinforcement leads to an increase in the shear resistance [8,21,30]. Reinforcement located in the vicinity of the joint reduces the crack development and thus the concrete softening effect [20]. However, as some studies demonstrated, the reinforcement ratio does not have a meaningful effect on the cracking load (when the adhesion breaks) [23]. On the other hand, the amount of transverse reinforcement determines the post-peak behaviour and at high reinforcement ratios (according to various studies and depending on the surface roughness, *ρ_s_* > 0.5% [23], *ρ_s_* = 0.99% [11]—very rough interface), a further increase in load is possible. This is, however, obviously related to the significant slip. The method of installing the transverse reinforcement (casted with the base element or post-installed into drilled holes) does not have a significant impact on the load capacity of the interface, provided that the appropriate embedment depth is maintained—in the tests [23], the difference did not exceed 8%. Shear reinforcement began to contribute after cracking of the interface. In the tests [11,31], the stress in the reinforcement at cracking did not exceed approximately 50% of the yield strength. Similar conclusions regarding the contribution of the reinforcement in the post-peak phase can also be found in [10,19].

Increasing the roughness of the joint surface leads to an increase in the load-carrying capacity of interfaces between concretes cast at different times by as much as 50 or even 120% [11,16,24]. At the same time, the drop in load immediately after cracking is much lower in the case of very rough contacts, which is explained by the mechanical adhesion (Verhakungskohäsion) [11]. In elements with smooth contacts, the effect of frictional forces is much lower and the effect of dowel action is governing (for comparison, in contacts with a rough surface, the contribution of dowel action is estimated to be about 10 to 30% [8,11]). Mean peak height *R_pm_* and mean peak-to-valley height *R_z_* are the parameters that were found to best describe the effect of the substrate profile on the load capacity of the interface between concrete [16,24].

Experimental investigations confirmed the effect of the aggregate composition. In case of lightweight aggregate concrete (LWAC), the aggregate is the weakest component susceptible to cracking and crushing, which translates to lower shear resistance by about 5–35% [13,16] compared to ordinary concrete. The failure plane is then characterized by much smaller unevenness [13], which translates into a reduction of interlocking and friction, while the effect of particle size is very limited [3]. The research demonstrated a significant effect of the LWAC density, depending on the type of aggregate used and the related crushing resistance [14,15]. Similar effects were also observed in high-strength concretes up to 120 MPa [12], where aggregate also constitutes the weakest component of concrete. In elements made from recycled aggregate concrete (RAC), a decrease in the load capacity of the interfaces is observed, reaching even 20%, depending on the ratio of replacement of the natural with recycled aggregate [17,18].

The tests of composite beams [27,28,29] clearly show that apart from the adhesion conditions in the joint surface and the intensity of transverse reinforcement, the location of the contact at the cross-section height and the relationship between the stiffness of the base element (substrate) and the concrete overlay are important. Depending on the relation between the forces at cracking of the interface and at the shear crack formation, the failure may be initiated by cracking of the interface or proceed in the same way as in monolithic elements.

Based on the observations from the previous experimental investigations, the behaviour of the contacts between concretes cast at different times can be characterized as follows. Initially, only a very slight slip is recorded as the load increases. Cracking appoints breaking of the adhesive forces and thus the failure of non-reinforced interfaces. If shear reinforcement is applied, the connection still retains a certain load capacity, which, however, depends on the amount of the reinforcement. After cracking, a drop in the registered force is observed. If the reinforcement is close to the minimal one, the residual load capacity stabilizes at a constant level and results mainly from the dowel action. Failure is a consequence of the rupture of reinforcement, but the interface shows ductile behaviour. In case of contacts with a very high reinforcement intensity (which corresponds, for example, to the connection between the flange and the web in beams), it is possible to achieve a load capacity higher than at the moment of cracking. The failure may result from concrete crushing or debonding of the transverse reinforcement (in case of post-installed dowels). However, the activation of additional forces resulting from the effect of aggregate interlock and dowel action requires significant displacements of the contact surface, which may not be acceptable with respect to serviceability (appearance and durability).

### 1.2. Design Procedures

According to the existing EN 1992-1-1 [4] procedure, the design shear resistance at the interface between concrete cast at different times is defined as follows:

(1) $$v_{Rdi}=\underset{\mathrm{adhesion}}{\underset{︸}{cf_{ctd}}}+\underset{\mathrm{friction}}{\underset{︸}{\mu \sigma_{n}}}+\underset{\begin{array}{c} \mathrm{friction}\;(\mathrm{clamping}\;\mathrm{effect})\\ \mathrm{dowel}\;\mathrm{action} \end{array}}{\underset{︸}{\rho_{s}f_{yd}\left(\mu \;sin\alpha +cos\alpha \right)}}\leq 0.5vf_{cd},$$

where *c* is the factor reflecting adhesion forces (see Table 2), *μ* is coefficient of friction, *σ_n_* denotes stress acting perpendicular to the shear plane, (not more than 0.6*f_cd_*), *ρ_s_* is shear reinforcement ratio (inclined at an angle of α to the interface), *f_yd_* represents yield strength of the reinforcement, and *vf_cd_* corresponds to the compressive strength of the cracked concrete.

The simultaneous action of the adhesion, friction, aggregate interlock, and dowel action of the reinforcement is assumed.

Contrary to [4], in *fib* Model Code 2010 [32], two basic types of interface behaviour were distinguished—rigid and non-rigid. In case of rigid behaviour, the shear resistance depends mainly on the adhesion and friction forces:

(2) $$\tau_{Rdi}=\underset{\mathrm{adhesion}}{\underset{︸}{c_{a}f_{ctd}}}+\underset{\mathrm{friction}}{\underset{︸}{\mu \sigma_{n}}}\leq \tau_{Rd,max},$$

where *c_a_* denotes the coefficient for the adhesive bond (see Table 2), *μ* is the coefficient of friction, and *σ_n_* represents the compressive stress (lowest expected) resulting from an eventual normal force acting on the interface.

In case of the interfaces with non-rigid behaviour, when the possibility of slip in the contact plane is allowed, the load capacity shall be calculated as follows:

(3) $$\tau_{Rdi}=\underset{\mathrm{aggregate}\;\mathrm{interlock}}{\underset{︸}{c_{r}\sqrt[{3}]{f_{ck}}}}+\underset{\mathrm{friction}}{\underset{︸}{\mu \sigma_{n}}}+\underset{\mathrm{clamping}\;\mathrm{effect}}{\underset{︸}{\kappa_{1}\mu \rho_{s}f_{yd}}}+\underset{\mathrm{dowel}\;\mathrm{action}}{\underset{︸}{\kappa_{2}\rho_{s}\sqrt{f_{cd}f_{yd}}}}\leq v\prime f_{cd},$$

where *c_r_* denotes the coefficient for aggregate interlock effects at rough interfaces (see Table 2), *κ*_1_ is the interaction coefficient for tensile force activated in the reinforcement, *κ*_2_ is the coefficient for flexural resistance, *ρ_s_* denotes the ratio of shear reinforcement crossing the interface, and *v’* is the coefficient for the strength reduction of the compressed struts. The *κ*_1_ and *κ*_2_ coefficients (interaction factors) limit the load capacity of the reinforcement due to the complex stress state resulting from the simultaneous tension (clamping effect) and bending (kinking effect) of the rebars.

In the prEN 1992-1-1 [33] procedure, an additional distinction between reinforced interfaces in terms of their behaviour into rigid and ductile was made. In the first case, the shear resistance is to be calculated according to the Equation (4), assuming that the shear reinforcement yield (i.e., it is properly anchored—a connection of precast elements is indicated as an example). The combined action of the adhesion, aggregate interlock, and friction is assumed:

(4) $$\tau_{Rdi}=c_{v1}\frac{\sqrt{f_{ck}}}{\gamma_{c}}+\mu_{v}\sigma_{n}+\mu_{v}\rho_{s}f_{yd}\leq 0.25f_{cd}.$$

The Equation (4) also applies to non-reinforced interfaces (*ρ_s_* = 0).

In case of the joints with ductile behaviour (as an example, concrete overlay cast on reinforced concrete slabs is indicated, where cracks due to shrinkage may occur) the shear resistance at the interface is defined as follows:

(5) $$\tau_{Rdi}=c_{v2}\frac{\sqrt{f_{ck}}}{\gamma_{c}}+\mu_{v}\sigma_{n}+k_{t}\mu_{v}\rho_{s}f_{yd}+k_{f}\rho_{s}\sqrt{f_{cd}f_{yd}}\leq 0.25f_{cd}.$$

In Table 2 the coefficients used in the discussed standard procedures were listed. A very similar definition of the friction coefficients *μ* and the coefficients expressing the adhesion forces *c* can be seen. The differences in the values of *c_a_* and *c_v_*_1_ result from replacing the concrete tensile strength in [33] with the square root of the compressive strength, which forced the adhesion coefficient to be adjusted.

The surface classification used in EN 1992-1-1 [4] was based on a subjective assessment of their roughness. In the procedures [32,33], evaluation criteria were introduced based on parameters related to the surface profile, such as the mean profile depth *R_t_* or mean peak-to-valley height *R_z_*—see Table 3.

**Figure 2:**
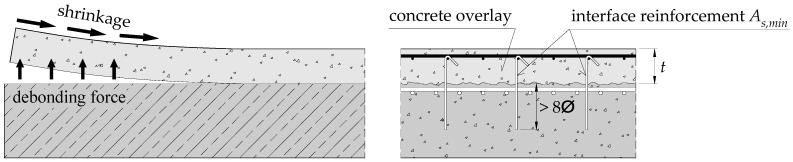
Idea of the minimum transverse reinforcement.

Draft of the standard prEN 1992-1-1 [33] clearly specifies the requirements for minimum reinforcement. Its cross-section should be selected in such a way to ensure transfer of tensile forces that may arise after cracking of the interface (see Figure 2):

(6) $$A_{s,min}=t_{min}\frac{f_{ctm}}{f_{yk}}.$$

where *t_min_* is a smaller value of the thickness of new and old concrete layer, and *f_ctm_* denotes tensile strength of the respective concrete layer.

The *fib* Model Code 2010 [32] additionally requires us to provide the minimum interface reinforcement, which should guarantee the transfer of shear forces by dowel action and aggregate interlock mechanisms, and ensure ductile behaviour of the connection (ultimate slip up to 0.5–1.5 mm):

- in beams *ρ_s,min_* = 0.20 *f_ctm_*/*f_yk_* > 0.1%,
- in slabs *ρ_s,min_* = 0.12 *f_ctm_*/*f_yk_* > 0.05%.

According to the ACI 318-19 [34] standard, the shear resistance is determined assuming that the reinforcement crossing the shear plane yields. Depending on the method of surface preparation and the type of contact (in monolithic concrete or between concretes cast at different times), the resistance is given as follows:

(7) $$v_{u}=\lambda \cdot \mu \rho_{s}f_{yd}\leq v_{u,max},$$

where $\lambda =\{\begin{array}{l} 1.0\;\;\;\mathrm{for}\;\mathrm{NWC}\\ 0.85\;\;\mathrm{for}\;\mathrm{SLWC}\\ 0.75\;\;\mathrm{for}\;\mathrm{ALWC} \end{array}$; $\mu =\{\begin{array}{l} 1.7\;\;\mathrm{monolithic}\;\mathrm{joint}\\ 1.4\;\;\mathrm{artificially}\;\mathrm{roughened}\;\mathrm{joint}\\ 1.0\;\;\mathrm{non}-\mathrm{prepared}\;\mathrm{joint} \end{array}$ and

$$v_{u,max}=\min\{\begin{array}{l} 0.2f_{c}^{\prime }\\ 3.3+0.08f_{c}^{\prime }\;\;\mathrm{for}\;\mathrm{NWC}\\ 5.5\;\mathrm{MPa}\;\;\;\;\mathrm{for}\;\mathrm{LWAC} \end{array}$$

The parameter *μ* in Equation (7) is not the friction coefficient in the literal sense of the word and also expresses the effects resulting from aggregate interlock and dowel action. The coefficient *λ* takes into account the aggregate composition. Depending on the lightweight aggregate content, a different reduction of the ultimate shear stress (from 15 to 25%) is expected in relation to ordinary concrete. These recommendations reflect the results of the tests by Mattock et al. [13].

AASHTO [35] recommendations, unlike [34], take into account the additional adhesion forces. Contrary to [4], a constant value of adhesion has been introduced, which is related only to the method of the surface preparation—see Table 4. The values of friction coefficients *μ* correspond to unprepared or deliberately roughened surfaces according to [34]. The ultimate shear stress at the interface is equal to

(8) $$v_{u}=c+\mu \rho_{s}f_{yd}\leq \min\{\begin{array}{c} K_{1}\cdot f_{c}\\ v_{u,max} \end{array},$$

where *c* denotes adhesion factor, *μ* is friction factor, *K*_1_ means factor reflecting fraction of concrete strength available to resist interface shear, and *v_u,max_* is limiting interface shear resistance specified, including the possibility of breaking or crushing of the aggregate.

## 2. Materials and Methods

### 2.1. Test Programme and Test Setup

**Figure 3:**
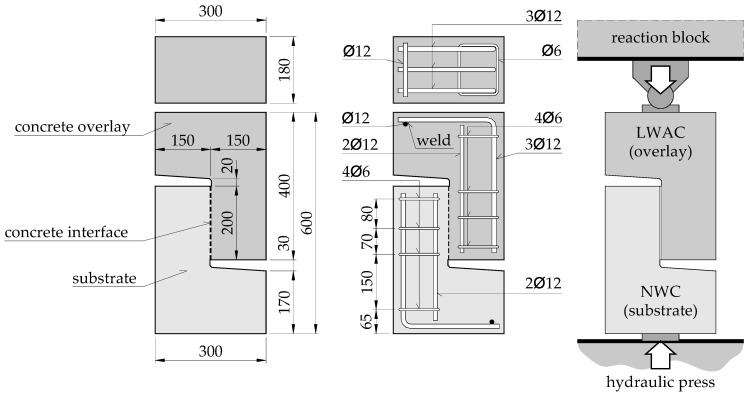
Details of the test specimens and the test setup (all dimensions in mm).

**Figure 4:**
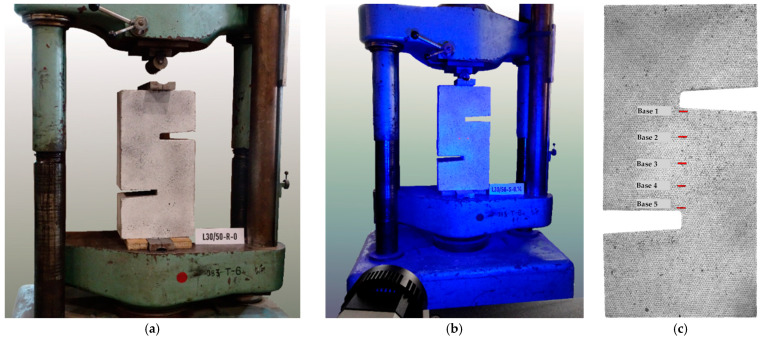
View of the specimen: (**a**) In the test setup before start of the test, (**b**) during measurements of the surface deformations by DIC, (**c**) detail of the surface pattern with computational mesh and bases for deformation measurements.

The presented study included a total of 12 push-off elements grouped in two research series. The test specimens consisted of two identical L-shaped parts, formed in successive stages and connected in the central part. The shape of the models is shown in Figure 3. The contact (interface) area was 180 × 200 = 36,000 mm^2^. The tests were carried out in a press stand with a maximum pressure of 1000 kN, enabling force control. The elements were placed in such a way that the axis of force application coincided with the contact surface. In order to ensure even pressure, the load was applied on 50 mm thick steel plates. The reaction block was equipped with a steel “knife” and roll bearing to enable free positioning of the upper steel plate—see Figure 4. Each time, the specimens were placed in such a way that the part made of lightweight aggregate concrete (overlay) was located at the top.

In order to determine the possible differences in the interface behaviour resulting from the type of overlay, two series of elements were made, differing only in the concrete overlay used. In both series, the overlay was made of LWAC, but with a different target strength class. In each test series, the following were differentiated: The method of surface preparation (smooth or mechanically roughened), affecting the roughness (expressed as the average profile depth *R_t_*, estimated according to Equation (10)), and the interface reinforcement (transverse reinforcement ratio *ρ_s_*). The test program is presented in Table 5. The following systematic description of the test specimens has been adopted: The letter denotes the type of concrete overlay (L—lightweight aggregate concrete), the following numbers indicate the target strength class of the base concrete and the concrete topping, the next letter is related to the description of the contact surface (S—smooth, R—rough), while the last number indicates the transverse reinforcement ratio. Elements without shear reinforcement were duplicated (0 and 0 bis).

### 2.2. Materials

The parts of the elements representing the base were made of normal, ready-mixed concrete with the designed compressive strength class C25/30. The fine aggregate was natural sand (fraction 0/2), while the coarse–granite crushed aggregate of fraction 2/8. The overlay was made of lightweight concrete with “Certyd” aggregate, which is a product of fly ash sintering as coarse aggregate (fraction 4/9), and natural sand (fraction 0/2) as fine aggregate. The lightweight aggregate used is characterized by a relatively high resistance to crushing above 5.0 MPa (for comparison, in case of LECA, it is 0.75–1.0 MPa). The composition of the mixtures was selected to obtain concrete classes LC25/28 (L30/30 series) and LC30/33 (L30/50 series) and density class 1.8 according to EN 206 [36]. CEM I Portland cement was used in all concrete mixes. Lightweight aggregate concrete from one batch was used in all elements of a given test series. Concrete composition is presented in Table 6.

The tests of the base concrete were carried out after 35–149 days after casting and also on the day of testing the composite elements—i.e., after 287 days (L30/50 series) and 350 days (L30/30 series). Due to the time that has elapsed since the day of casting, no significant differences in the characteristics of the concrete were found (coefficients of variation below 5%). The tests of the overlay concrete were carried out each time on the test day of the composite elements. The strength properties were determined on cylinders ∅150 mm (compressive strength *f_cm_* and secant modulus of elasticity *E_cm_*) and cubes of a side length of 150 mm (tensile strength by splitting *f_ctm,sp_*). The results obtained are presented in Table 7.

**Figure 5:**
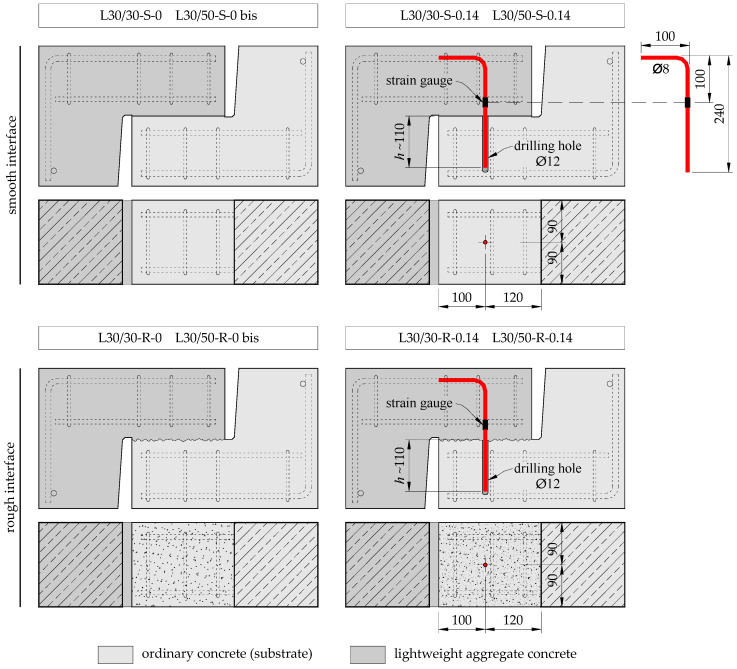
Details of the test specimens (all dimensions in mm).

The reinforcement was made of B500C grade steel. L-shaped parts of the specimens were reinforced longitudinally with 3∅12, mechanically anchored with a welded bar. Transverse reinforcement consisted of 4 closed stirrups ∅6, concentrated in the contact area. In two models of each series, characterized by different roughness of the contact surface, shear reinforcement in the form of ∅8 bar applied—see Figure 5. Based on the tests of the steel samples, the characteristic parameters of the shear reinforcement were determined: Yield strength *f_ym_* = 598.3 MPa, tensile strength *f_um_* = 642.8 MPa, and modulus of elasticity *E_s_* = 193.9 GPa. Details of the reinforcement of the test specimens are presented in Figure 3 and Figure 5.

The cross-section of the shear reinforcement was selected to obtain the interface reinforcement ratio typical for slabs reinforced with concrete topping and at the same time to enable ductile behaviour in accordance with the requirements of *fib* Model Code 2010 [32] (*ρ_s_* > 0.05% and at the same time *ρ_s,min_* = 0.12 *f_ctm_*/*f_ym_* = 0.12·3.49/598.3 = 0.07%):

(9) $$\rho_{s}=\frac{A_{s}}{b\cdot t}=\frac{50.2}{180\cdot 200}=0.14\%,$$

### 2.3. Preparation of the Test Specimens

**Figure 6:**
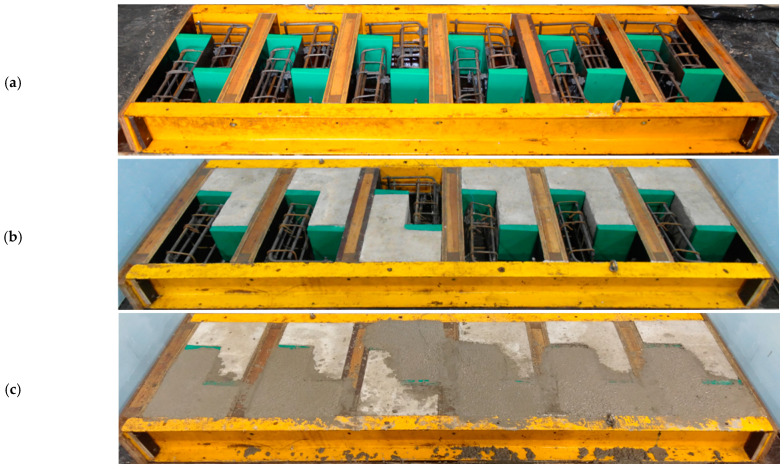
Subsequent stages of casting test specimens: (**a**) Placing of the reinforcement, (**b**) casting of parts constituting the substrate, (**c**) placing concrete overlay.

In order to reflect the conditions encountered in real structures subject to strengthening, the specimens were made in the following steps, as shown in Figure 6:

1. Casting of the substrate parts and their subsequent conditioning in the laboratory,
2. preparation of the surface of the interface by gentle grinding (to remove the excess of cement laitance) or graining, carried out approximately 6 months after casting the base elements,
3. drilling holes and injecting of the post-installed reinforcement (in selected elements), and
4. casting concrete overlay.

In order to control the quality of the surface roughness, the sand path method was used. The surface roughness was assessed by measuring the diameter of the circle *D* resulting from the uniform distribution of a precisely defined volume (*V* = 10 cm^3^) of fine-grained sand (*D_g_* < 0.12 mm), so that it filled any irregularities. The measurements were repeated two or three times, and the average value was taken as the final result. The mean surface roughness was determined according to the following relationship:

(10) $$R_{t}=\frac{1273V}{D^{2}},$$

where *V* denotes the volume of sand used in the test, equal to 10 cm^3^, and *D* is diameter of the circle formed after the sand is evenly distributed.

**Figure 7:**
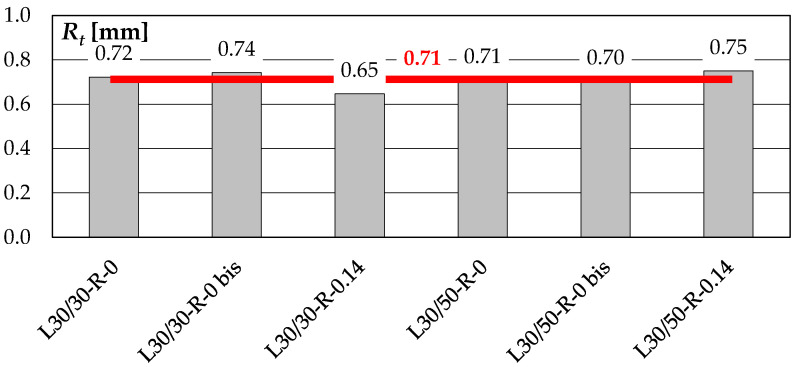
Roughness of the interface surface of the test specimens.

The results of the calculations made on the basis of the measurements are summarized in Figure 7. The roughness of the surfaces was similar and equal to 0.71 mm on average, what corresponded to mean depth of the grooves of 1.4 mm. The grained surface, however, should be classified as smooth according to the provisions of *fib* Model Code 2010 [32] and prEN 1992-1-1 [33], as *R_t_* < 1.5 mm.

**Figure 8:**
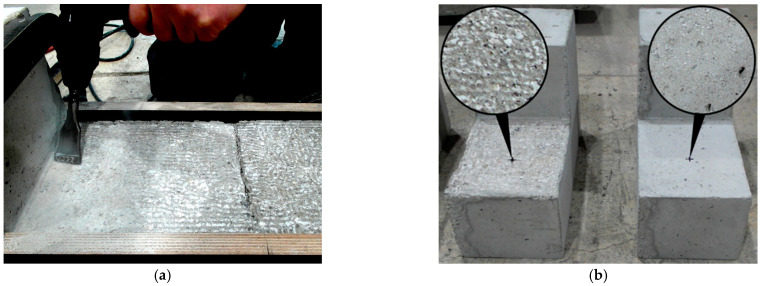
Surface preparation: (**a**) Milling with a chisel, (**b**) view after by milling and soft grinding.

In order to obtain the desired roughness of the substrate, the surface was prepared with a concrete chisel enabling formation of longitudinal grooves—see Figure 8a. By moving the chisel in both perpendicular directions, a surface with the assumed profile was obtained. Due to concerns regarding the bonding of the base and the concrete overlay, it was decided to polish the contact surface of the S-series specimens as well. This treatment was aimed solely at removing the cement laitance and did not significantly increase the surface roughness. Figure 8b shows the surfaces of the substrate elements. Before casting the concrete overlay, the base elements were stored in water for 12 h in order to reduce the water absorption from the new concrete and thus early cracking of the interface. After placing the concrete overlay, the elements were cured for about 7 days by systematic wetting and covering with foil to reduce drying.

**Figure 9:**
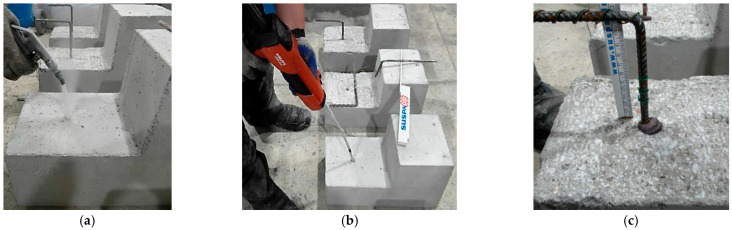

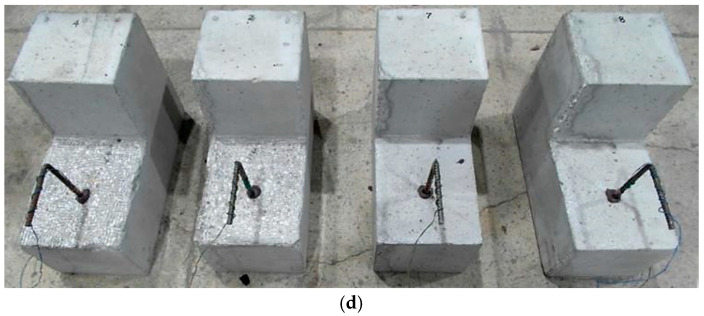
Subsequent stages of preparation of the interface reinforcement: (**a**) Cleaning of bored holes with compressed air, (**b**) application of the adhesive resin, (**c**) installing of the deformed rebars, (**d**) curing of the resin.

In two specimens of each series, additional shear reinforcement was provided. It was injected into the holes drilled in the base elements, after the surface had been roughened. Holes with diameter of 12 mm and a depth of about 115 mm were made, each time carefully cleaned, and any contamination was removed from them with compressed air (Figure 9a). Then, the holes were filled to about ½ its depth with adhesive material based on the hybrid resin (Figure 9b), and then the reinforcing bars were installed. The bars were mounted in a twisting motion so that the adhesive evenly filled the space between the side of the hole and the rebar. The excess of the adhesive flowed out from the opening (Figure 9c). The installed reinforcement was showed in Figure 9d. The embedment depth was *h_ef_* = 110 mm > *h_ef,min_* = 80 mm. After applying the reinforcement, the elements were left in the laboratory until the curing process of the resin was completed.

### 2.4. Testing Procedure and Measuring Equipment

After the element was placed and centred in the test setup, the load was applied. Initially, the load was increased incrementally by 2.5 or 5 kN at each step. Starting from a load level of 25–30 kN, the load was applied in a uniform manner until the failure. Each test lasted about 5–10 min. During the tests, surface deformations were measured using the Digital Image Correlation (DIC) system GOM Aramis. It consisted of two high-resolution cameras of 2752 × 2200 pixels, enabling the recording of 25 frames/second, connected with a controller and computer equipped with software that allows us to track 3D surface deformations using the triangulation method. The tracked area covered the entire front face of the specimen (300 × 600 mm). To provide the measurements, it was necessary to apply a pattern in the form of black dots of different diameter. During the test, it was possible to track the crack development in real time, as well as the displacement of any points on the surface by indicating virtual benchmarks and extensometers. The bases, on which the slip and crack width were recorded, were evenly distributed along the contact length in five levels, approximately every 40 mm. In addition, in case of the elements with reinforced interface, strains of the rebars were measured in the vicinity of the contact surface in points marked in Figure 5. For this purpose, 5 mm electrofusion strain gauges glued on rebar with cyanoacrylate adhesive and connected with a data acquisition system were used. The wire was routed perpendicular to the shear plane and outside surface of the rebar to not affect bond conditions.

## 3. Results

### 3.1. Crack Pattern

**Figure 10:**
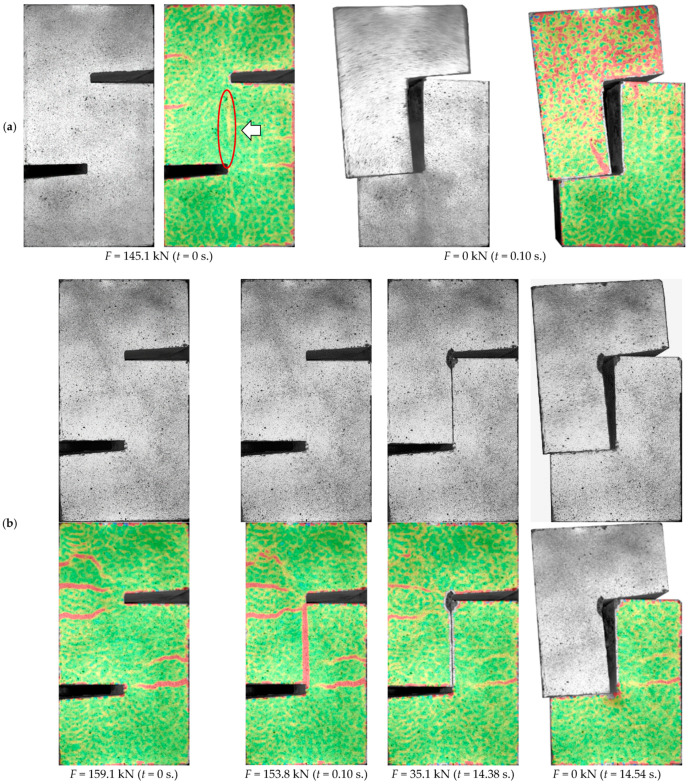
View of the surface of: (**a**) L30/50-S-0, (**b**) L30/50-S-0.14—crack formation at subsequent load levels and major strains (values in parentheses indicate the time measured from the moment of reaching the maximum load).

The real-time analysis of deformation fields (see Figure 10) made it possible to capture the moment of appearance of the first cracks. Initially, they resulted from bending and were visible on the edges of the specimens.

Cracks at the interface were registered at higher loads levels. In case of the specimens without shear reinforcement occurrence of the interface crack was synonymous with exhaustion of load carrying capacity because within fractions of a second (~0.1 s), failure manifested with a rapid detachment of the concrete block was observed. An example of the crack development and destruction process of L30/50-S-0 model, typical for specimens without shear reinforcement, was presented in Figure 10a.

The introduction of the reinforcement crossing the contact surface resulted in a qualitative change in the failure mode. Although a drop in load was recorded immediately after cracking, the connection was still able to maintain a certain residual load capacity, which was initially due to the aggregate interlock and in the final phase mainly from the dowel action of the reinforcement. The cooperation of the transverse reinforcement allowed for a ductile failure. The rupture of the contact was significantly delayed and was accompanied by a clear slip in the shear plane, which is visible in Figure 10b. The crack development was typical for all elements, regardless of the strength of the concrete overlay used.

### 3.2. Slip and Crack Opening

**Figure 11:**
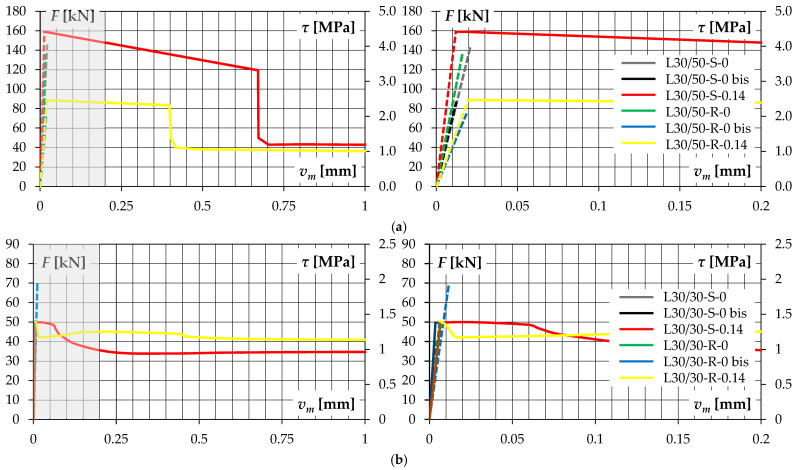
Applied force—slip relationships for test specimens of: (**a**) L30/50 series, (**b**) L30/30 series.

In Figure 11, diagrams illustrating the relationship between the load and the slip (mutual displacement of the contact surfaces) were presented. The displacements were determined by using DIC, according to measurements in five bases (virtual extensometers) evenly distributed along the contact length, showed in Figure 4c. Particularly in the initial phase of the test (before cracking), very small slip was recorded, therefore it was decided to enlarge this range so that it was possible to trace displacements that were not visible at large scales (the range marked in grey in Figure 11).

The average slip *v_m_* at interface cracking was very small and did not exceed about 0.02 mm. A certain variability of the recorded values was observed in the initial range, which resulted from the technical possibilities and the accuracy of the applied measurement technique. For this reason, the course of slip as a function of load up to the crack formation was shown with a dashed line to indicate the presumed nature of this relationship. Two or even three times higher values were recorded for the L30/50 series elements—the slip *v_m_* was about 0.012–0.021 mm, while in case of the L30/30 specimens with LWAC overlay of lower strength they amounted only about 0.004–0.012 mm—see Table 8. However, attention should be paid to the different destructive forces in both series.

The installed transverse reinforcement was insufficient to maintain the load capacity after cracking at a similar level. However, it made it possible to change the failure mechanism to be more ductile. The joint was characterized by a certain residual load capacity *F_res_*, which was about 27–35 kN, while the failure related to rupture of the reinforcement occurred at the slips *v_max_* of 7 to even 12 mm.

**Figure 12:**
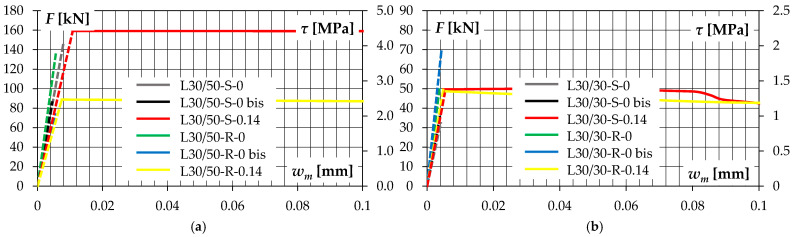
Applied force—crack width relationships for test specimens of: (**a**) L30/50 series, (**b**) L30/30 series.

The width of the microcracks tracked on the surface of the elements immediately before failure or their transformation into macrocracks did not exceed about 0.01 mm. Slightly higher values were recorded for elements of the L30/50 series—*w_m_* = 0.005–0.011 mm—see Figure 12. For elements of the L30/30 series, the microcrack widths were in range of *w_m_* = 0.004–0.008 mm. At the moment of interface cracking in the elements with transverse reinforcement, a sudden opening of the cracks to about 0.1–0.3 mm was observed. These cracks were already visible to the unaided eye. At the time preceding failure, their widths were approximately *w_max_* = 1.7–1.8 mm in elements with smooth contact surface and 2.0–2.2 mm in elements with rough contact surface.

### 3.3. Strains of the Shear Reinforcement

**Figure 13:**
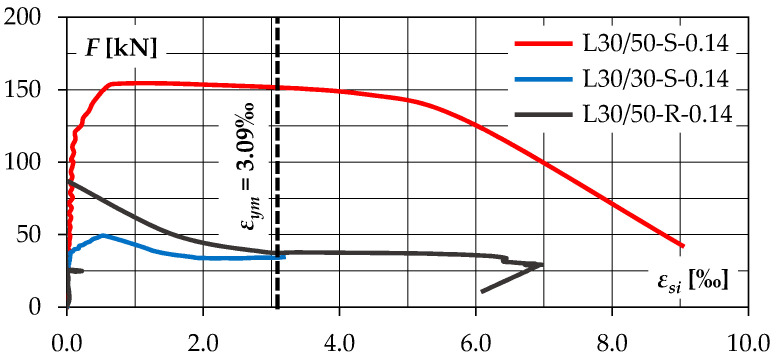
Strains of the interface reinforcement.

The results of strains measurement of the reinforcement crossing the shear plane were presented in Figure 13. Due to the initial failure of the strain gauge, it was impossible to track strains in the case of the L30/30-R.014 specimen. In the initial phase of the test, the strains were close to zero. In case of the element with a rough interface (L30/50-R-0.14), a noticeable increase in deformation occurred only after reaching the maximum load, when the interface was cracked. Within one second, a change in deformation from −0.032% to 1.531% was registered, which was accompanied by a rapid decrease in loads from 87.0 to 57.5 kN. On the other hand, in elements with smooth interface (L30/30-S-0.14 and L30/50-S-0.14), a gradual increase in strains preceding cracking of the interface was observed. These changes, however, were not significant, and when the maximum load was reached, the stress in the reinforcement did not exceed about 20% of the yield point—see Table 9. These observations are consistent with the results of previous experimental studies, including Randl and Wicke [11] and Fang et al. [19], proving the non-simultaneous cooperation of the adhesion and mechanisms related to the transverse reinforcement.

In all cases, after cracking, an intensive increase in strains of the reinforcement was recorded. Initially, it was, to a very limited extent, a consequence of the crack opening resulting from aggregate interlock. However, the installed reinforcement was insufficient to ensure effective restraint. As a result, a sharp increase in the mutual displacement of the contact surface (slip) was observed after cracking. In this phase, the load capacity was governed only by the dowel action, which was manifested by a very intense increase in deformations at the initially constant and then decreasing load *F* in the range of 30–40 kN.

### 3.4. Failure Mode

**Figure 14:**
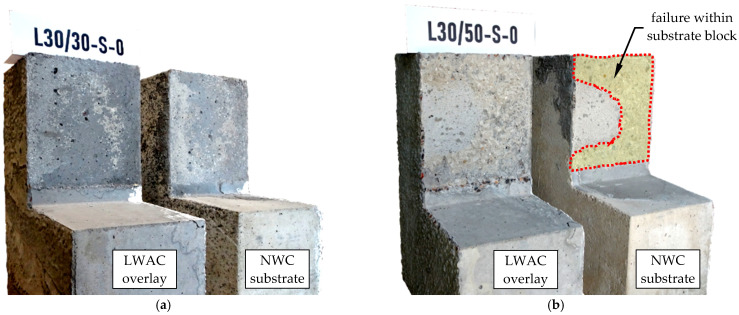
View of the test specimens with smooth interface after failure: (**a**) L30/30-S-0, (**b**) L30/50-S-0.

Figure 14 shows the details of the contact surface between old and new concrete of L30/30 and L30/50 series specimens with a smooth interface conditions (S). In case of elements where the compressive strength of the substrate concrete was higher than the strength of the overlay, the destruction was related to the detachment of the concrete topping. The shear plane crossed the subsurface layer of hardened cement grout—see Figure 14a. The failure resembled in a way the "unsticking" of the block made of LWAC, because the polished surface of the substrate was exposed. The compressive strength of the substrate concrete was in this case by over 20% higher than that of LWAC overlay. When considering the tensile strength, the difference was even higher and exceeded 40%, which resulted in a limited specific adhesion. Taking into account the low mechanical adhesion resulting from the surface preparation method (soft grinding), it was a natural thing that failure occurred within LWAC layer.

Although the contact surface of L30/50-S-0 specimen was prepared smooth, the failure plane had a rough texture and crossed the substrate block over a greater part of the contact area (this area was marked with yellow in Figure 14b). The reasons for this should be seen in the higher strength of the LWAC overlay, which also resulted in increased specific adhesion. It is worth noting that while the difference in the compressive strength of NWC and LWAC was almost 30%, in relation to the tensile strength it was only 15%. In this case, ordinary concrete turned out to be the weaker component of the composite, but taking into account the possible differentiation of the strength properties of the overlay concrete, local detachment of the LWAC thin layer could also be expected.

**Figure 15:**
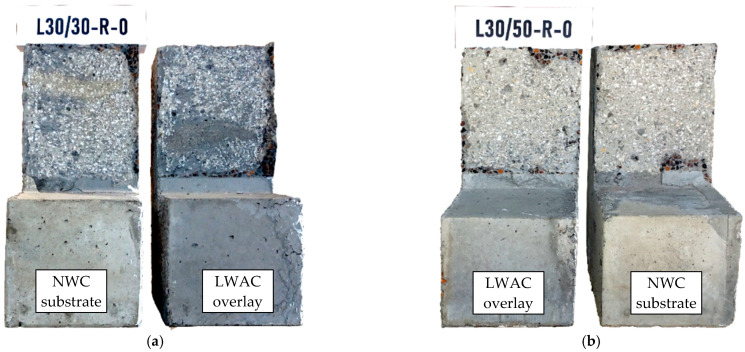
View of the test specimens with rough interface after failure: (**a**) L30/30-R-0, (**b**) L30/50-R-0.

In the case of elements where the contact surface was deliberately roughened by graining (R), the failure plane always passed through the substrate—regardless of the strength of the overlay concrete, which is shown in Figure 15.

**Figure 16:**
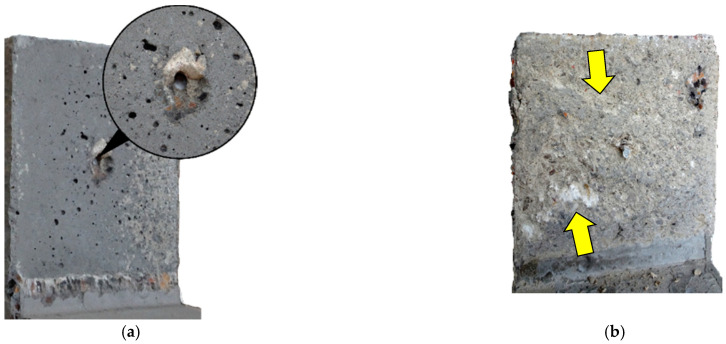
View of the test specimens with interface reinforcement after failure: (**a**) L30/30-S-0.14, (**b**) L30/50-S-0.14.

The reasons for this can be found in the invasive method of surface preparation. When removing the upper layer of concrete cover from the substrate with an impact graining hammer, the internal structure of the concrete could be damaged and microcracks could formed, which affect the reduction of the effective strength of the sub-surface concrete layer. This was also reflected in the destructive forces, which is discussed in more detail in the next section. The installation of shear reinforcement did not affect the shape of the failure plane, which was dependent primarily by the method of surface preparation. On the other hand, abrasion (crushing) of the aggregate related to the interface slip connected with the clamping effect was observed (see Figure 16). In the vicinity of the injected rebar, local crushing of concrete resulting from local pressure due to the kinking effect was also visible.

### 3.5. Experimental Loads

**Figure 17:**
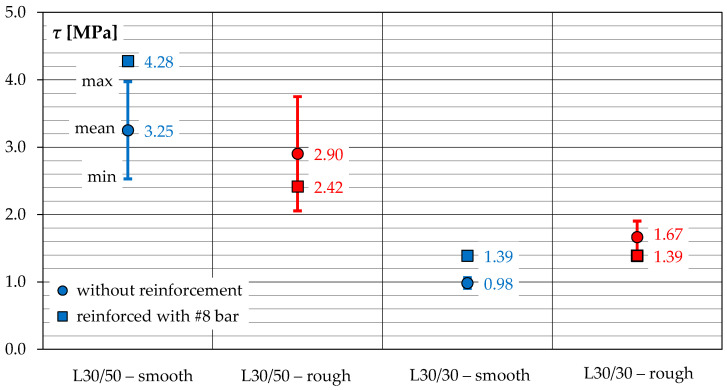
Shear stress at peak load corresponding to specimens of the subsequent test series.

Figure 17 summarizes the maximum shear stress *τ*, corresponding to the maximal loads registered during the individual tests. In case of specimens without shear reinforcement, the mean of the two results (marked as a point) and the difference between the individual results (min/max) were indicated. It can be seen that change in the overlay concrete strength from 51.6 to 32.2 MPa was accompanied by a decrease in the shear stress *τ*. For elements without shear reinforcement, the difference was over 230% and about 75%, in case of smooth and rough contact surfaces, respectively. In case of elements with post-installed ∅8 bar, the difference was equal to about 210% and 75% in cases of smooth and rough contact.

It is worth noting that surface roughening by mechanical graining did not always lead to an increase in the load carrying capacity. Despite the fact that the surface was rougher, as mentioned earlier in Section 2.3, in case of the L30/50 series specimens, load carrying capacities were found to be lower by approximately 10 or even 43% with respect to the elements with smooth contact surface, for elements without and with shear reinforcement, respectively. The reasons should be seen in the local damage of the concrete structure, related to the invasive method of surface roughening. As a result of the impact action, microcracks formed, which reduced the strength of the subsurface concrete layer. It was reflected in the failure mode, because the failure plane crossed the substrate layer. It should be noted that the concrete overlay in the L30/50 series specimens had by 16% higher tensile strength than the substrate concrete. Therefore, the load capacities of the composite elements were primarily governed by the strength characteristics of the "old" concrete, which could additionally be lowered as a result of mechanical surface treatment.

In case of the L30/30 series specimens, when the concrete overlay was characterized by a lower strength than the substrate, the beneficial effect of mechanical surface preparation was noticeable. Specimens with a deliberately roughened surface were characterized by an average of almost 70% higher load carrying capacities than elements with smooth contact. Thus, the tendency was the opposite to that in the L30/50 series, what can be attributed to the fact that concrete overlay had by almost 40% lower tensile strength than the substrate (*f_lctm_*/*f_ctm_* = 0.61). Therefore, the strength of LWAC overlay was decisive for the load carrying capacities of smooth contacts. Increasing the contact area by graining affected primarily the increase of mechanical adhesion, due to the penetration of the mortar into the pores and unevenness of the substrate [22].

By analysing the test results of both series, it can be concluded that post-installed reinforcement in form of a single ∅8 rebar allowed to significantly increase the load capacity of elements with smooth contact—this change was about 30 and 40%, for the L30/50 and L30/30 series, respectively. However, this reinforcement turned out to be ineffective in case of deliberately roughed surfaces. In elements with smooth contacts, the gradual joining of the reinforcement was recorded even before reaching the maximum load, while in the elements with rough contact, tensile forces were registered only after interface cracking (see Figure 13). It can be presumed that in this case, mechanical and specific adhesion were dominant. Only after adhesion was broken at cracking, was the load transmitted on the transverse reinforcement. In both test series, the load carrying capacities of the specimens with a rough contact and reinforced with the ∅8 bar were about 17% lower compared to models without reinforcement.

**Figure 18:**
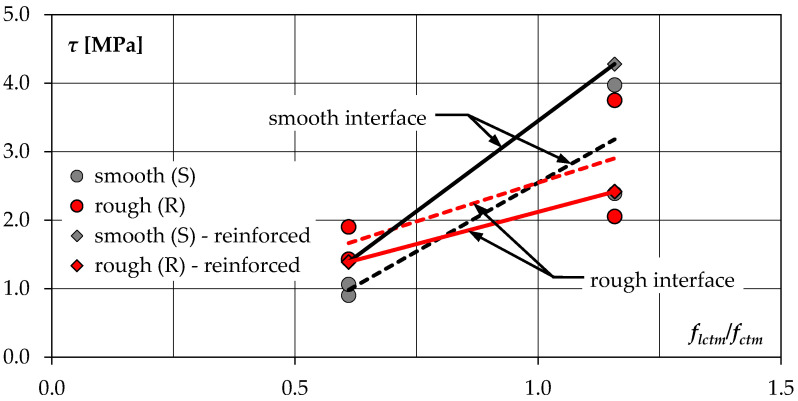
Relation between shear stress at peak load and concrete tensile strength ratio (solid lines—specimens with shear reinforcement, dashed lines—specimens without reinforcement).

Figure 18 shows the maximum shear stress as a function of the overlay and substrate concrete strength ratio *f_lctm_*/*f_ctm_*. A higher influence of the relationship between the strengths of both concretes is observed in case of elements with smooth contact. The parallel course of the regression lines for specimens with reinforced (continuous lines) and non-reinforced (dashed lines) interface and surface prepared in the same way proves that the decisive factor was the contribution of the adhesion forces, which were a function of the overlay and substrate concrete strengths.

**Figure 19:**
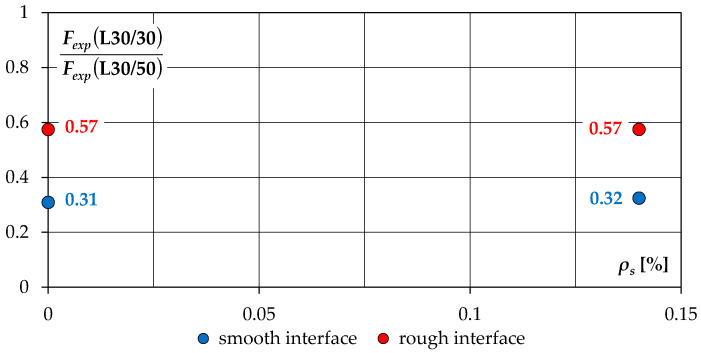
Comparison between experimental loads of the L30/30 and L30/50 series specimens.

Figure 19 shows the results of the comparison between load carrying capacities of the L30/30 and L30/50 series specimens, in which the substrate was the same strength.

It can be noticed that, regardless of the shear reinforcement ratio *ρ_s_*, similar relations between the ultimate loads of both test series elements with the same surface conditions were obtained. The highest differences were noted in the case of elements with smooth contact—the load capacity of the L30/30 series elements was almost 70% lower compared to the L30/50 series models. In case of specimens with a roughened interface, the difference was lower and amounted to 43%. The reasons for this can be seen in the high differentiation of the tensile strength of LWA concretes—in the L30/30 series, it was almost 50% lower than in the L30/50 series. This also translated into lower adhesive forces, which was especially noticeable in the case of smooth contacts.

### 3.6. Mechanism of Shear Transfer

Based on the strain measurements of shear reinforcement, an assessment of the mechanisms related to the contribution of concrete (adhesion forces, aggregate interlock) and reinforcement (clamping effect, dowel action) on the load carrying capacity was made. The steel contribution was established assuming that bars acted mainly as steel dowels. It was calculated taking into account the theoretical load capacity of a bar subjected to pure shear cutting:

(11) $$F_{s,v}=\frac{F_{s}}{\sqrt{3}}=\frac{\varepsilon_{si}E_{s}\cdot A_{s}}{\sqrt{3}}\leq \{\begin{array}{c} \frac{A_{s}f_{ym}}{\sqrt{3}}=17.3\;\mathrm{kN}\;\mathrm{if}\;\varepsilon_{ym}<\varepsilon_{si}\leq \varepsilon_{yh}\;\\ \frac{A_{s}f_{um}}{\sqrt{3}}=18.6\;\mathrm{kN}\;\mathrm{if}\;\varepsilon_{yh}<\varepsilon_{si}\leq \varepsilon_{um} \end{array},$$

where *ε_si_* denotes strains of shear reinforcement, *ε_yh_* corresponds to steel strains at the end of the yield plateau, *ε_ym_* is strain at the yield point (3.09%), *ε_um_* is strain at peak stress (~4%), *f_ym_* is yield point, *f_um_* is tensile strength, and *A_s_* expresses the cross section of the rebar (50.2 mm^2^).

**Figure 20:**
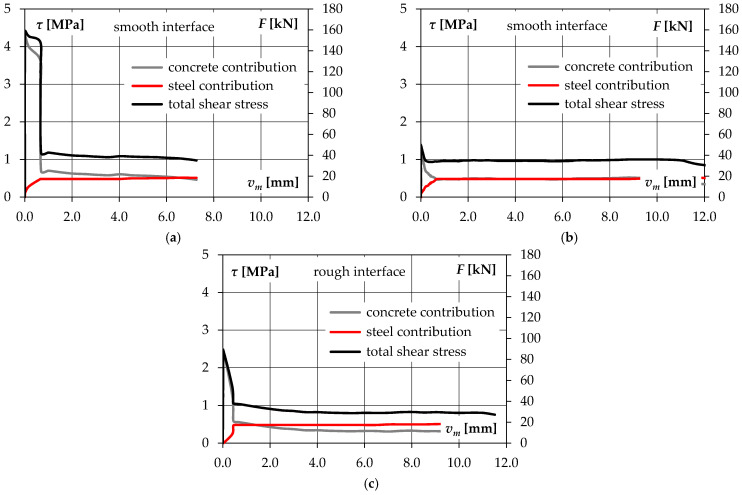
Shear transfer components as a function of interface slip: (**a**) L30/50-S-0.14, (**b**) L30/30-S-0.14, (**c**) L30/30-R-0.14.

Applying the philosophy of the shear-friction theory, a similar, maximum contribution of the shear reinforcement would be obtained by assuming the equivalent coefficient of friction *μ* = 1/√3 = 0.58 ≈ 0.6 (which corresponds to smooth surfaces according to EN 1992-1-1 [4]). The load capacity related to the concrete contribution was calculated as the difference between the applied load *F* and the steel contribution *F_s_*: *F_c_* = *F* − *F_s_*. The contribution of the indicated mechanisms as a function of interface slip is shown in Figure 20.

By analysing the obtained graphs, it can be stated that the mechanism related to the concrete contribution is initially dominant. The reinforcement becomes noticeably active only after cracking, which, however, is associated with a decrease in the concrete contribution. It is worth noting that the load capacity corresponding to yielding of shear reinforcement is achieved with a slip of approximately 0.5 mm (rough surface) and 0.7 mm (smooth surface). It can indicate a not as high effect of the aggregate interlock, which would result in a much earlier yielding of the reinforcement in the case of the L30/50-R-0.14 element with a rough surface.

**Figure 21:**
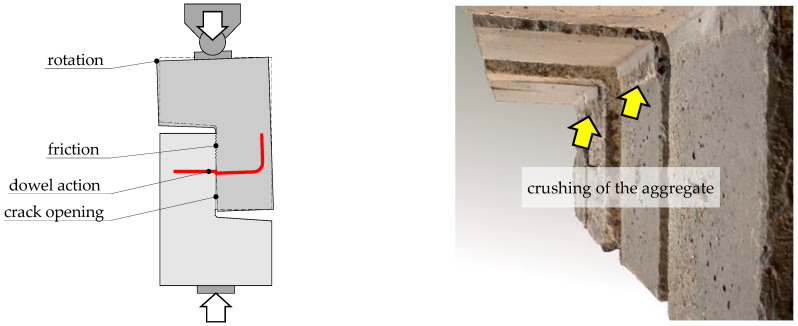
Frictional stress and aggregate crushing resulting from local pressure of the concrete block.

The plotted dependencies also show that the dowel action of the reinforcement was not the only mechanism that acted in the post-peak phase. In case of all of the specimens, the load capacity was about twice as high as it would result from the contribution of shear reinforcement only. The reasons for it can be found in the frictional forces that occurred on the contact surface. Based on the analysis of the measurements of the crack width along the contact length, it was possible to conclude that the crack was opened more strongly in the lower part of the interface. The reinforcement located in the centre of the contact area forced a slight rotation of one of the concrete blocks. This suggested local pressure in the upper part of the joint, which is schematically explained in Figure 21. This observation may be confirmed by the signs of aggregate abrasion.

## 4. Discussion of the Test Results

The results of the tests were compared with the procedures of the existing codes of practice: EN 1992-1-1 [4], prEN 1992-1-1 [33], AASHTO [35], and ACI 318-19 [34]. Due to the low reinforcement ratios (close to the minimum *ρ_s,min_*), the behaviour of reinforced joints was defined as “rigid” in accordance with the provisions of prEN 1992-1-1 [33], however, for comparative purposes, the load capacity corresponding to non-rigid behaviour was also calculated. All of the specimens consisted of two different types of concrete of various strength properties. When calculating the shear resistance, not only the strength of the weaker concrete (as stated in [4]) was taken into account, but also the theoretical shear stress corresponding to the strength of each concrete were determined. The lowest calculated shear stress was assumed as a final shear resistance. Such an approach was dictated, among others, by distinguishing between the rules of design of ordinary and lightweight aggregate concrete interfaces according to the AASHTO [35] and ACI 318-19 [34] procedures. The calculations were carried out taking into account the average values of the strength parameters of concrete and reinforcing steel, assuming all partial safety factors equal to 1.0.

**Figure 22:**
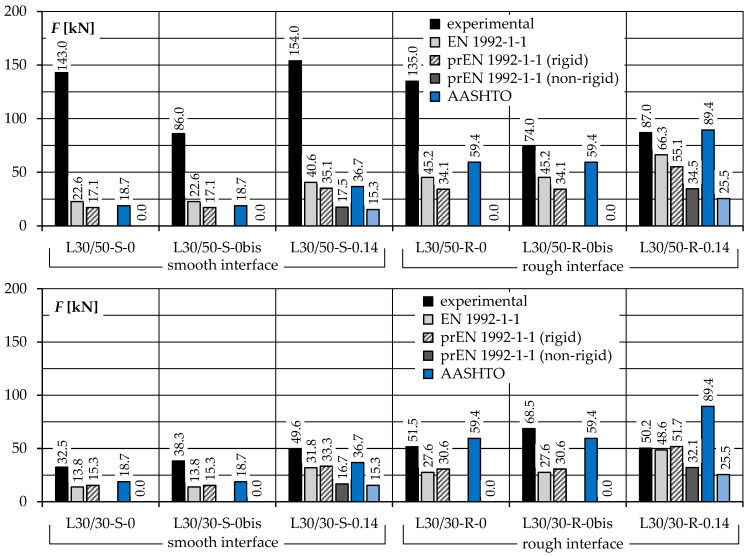
Comparison between experimental and predicted carrying capacities according to various design procedures.

The results of the calculations for the elements of the individual test series are shown in Figure 22. In the case of the specimens of the L30/50 series, the predictions of all considered design procedures *F_calc_* turned out to be lower than the experimental load carrying capacities *F_exp_*. This difference is especially noticeable for elements with smooth contacts—the ratio *F_exp_*/*F_calc_* is in the range of 4.6–10.1. However, in the case of elements with rough contacts, these differences were much lower—*F_exp_*/*F_calc_* = 1.5–3.4.

The theoretical load carrying capacities of all of the L30/30 series specimens were closer to the experimental ones—the calculated *F_exp_*/*F_calc_* ratios were equal to 1.7–3.2 and 0.9–2.0 for smooth and rough contacts, respectively. The predictions of the AASHTO [35] procedure turned out to be closest to the real load capacities of the L30/30 series models, however, in two cases these were overestimated. Contrary to EN 1992-1-1 [4] and prEN 1992-1-1 [33], the AASHTO procedure [34] provides for constant values of the coefficient expressing the adhesion forces, depending only on the surface classification. Thus, despite the significant differences in the strength of the overlay concrete in both test series (the difference of 19.4 MPa, i.e., almost 50% of the strength of the substrate concrete), the AASHTO [34] provided for the same load carrying capacities for corresponding elements of both L30/50 and L30/30 series.

The results of the calculations according to EN 1992-1-1 [4] and prEN 1992-1-1 [33] were similar, which arise from a similar definition of shear resistance in case of the interfaces with rigid behaviour. The slight differences in the experimental load capacities are due to different definitions of the adhesion forces, which, according to [4], are related to the tensile strength of concrete, while in [33] they are dependent on the square root of the concrete compressive strength.

The ACI 318-19 [34] standard turned out to be the most conservative. As the only one among all design procedures, it does not include the contribution of adhesion forces, conditioning the load capacity only on the shear-friction mechanism. Thus, the theoretical load carrying capacities of the elements without shear reinforcement were equal to 0. In case of the specimens with post-installed bars, the predictions were also very conservative (*F_exp_*/*F_calc_* = 2.0–10.1).

## 5. Conclusions

The paper presents results of the tests on 12 push-off specimens reflecting connection between concretes cast at different times. The main goal was to determine the load carrying capacity and behaviour of the elements in which the lightweight aggregate concrete constitutes the structural overlay. The results obtained in the study can be summarized as follows:

- Differences in failure mode and load carrying capacities depending on the method of surface preparation and the strength of the lightweight aggregate concrete overlay were found—the load capacities of the L30/50 series specimens were higher by about 40% and 70% compared to the corresponding models of the L30/30 series, in case of the roughened and smooth contacts, respectively;
- in elements with a smooth interface (S type models), the failure plane crossed the overlay concrete if it was characterized by a lower strength than the substrate (the failure resembled "peeling off" of a concrete block); however, if the strength of the overlay concrete was higher, then the failure plane was located within the subsurface layer of the substrate;
- in case of the elements with a deliberately roughened interface (R type specimens), the failure plane always crossed the substrate concrete, which can be explained by lower strength due to micro-cracks resulting from an invasive surface preparation method;
- the introduction of shear reinforcement in the form of post-installed ∅8 bars resulted in a change in failure mode from brittle to more ductile, but did not significantly affect the load capacity of the interface;
- after cracking, a drop in load was recorded, which means that the post-installed reinforcement used was insufficient to compensate for the loss of load carrying capacity resulting from breaking the specific adhesion forces, which proves their decisive influence at the low interface reinforcement ratios at the level of *ρ_s_* = 0.14%—due to the low carrying capacity of the shear reinforcement as well as limited strength of the lightweight aggregate (the tests demonstrated crushing and abrasion of aggregate grains) the resistance resulted from specific adhesion;
- the post-peak load capacity was approximately 23–31% and 65–67% of the maximum load, respectively, in case of L30/50 and L30/30 series specimens and resulted mainly from the contribution of the shear reinforcement (dowel action) and frictional forces;
- strain measurements of the shear reinforcement showed that it did not yield at interface cracking—in case of elements with a smooth surface, the steel stress accounted for about 18–23% of the yield point, while in case of an element with a surface roughened by graining, they were close to zero;
- an intense increase in steel strains was recorded only in the post-peak phase, when rebars acted mainly as dowels (dowel-action mechanism was decisive);
- it was found that most of the existing design procedures [4,33,34,35] allowed for a safe, but sometimes very conservative, estimation of the load carrying capacity of the interfaces between normal and lightweight aggregate concrete, cast at different times; the highest differences were noted in case of L30/50 series elements with smooth contact, when the experimental load capacities were even 10 times higher than the experimental ones;
- the discrepancies between the results of the tests and calculations resulted primarily from the insufficient description of the forces related to adhesion—in case of the AASHTO [35] procedure, the contribution of adhesion depends only on the method of surface preparation and is not related to the strength of concrete, while in the ACI 318-19 [34] procedure, based on the shear-friction theory, these forces are ignored and the theoretical load carrying capacities of the elements without shear reinforcement were equal to zero;
- despite a different design philosophy, adopted in prEN 1992-1-1 [33], where it is assumed that adhesion does not act simultaneously with aggregate interlock and dowel action effect, the slightly worse agreement between results of tests and calculations with respect to the EN 1992-1-1 [4] procedure was obtained—the mean *F_exp_*/*F_calc_* ratios were equal to 3.66 and 2.66, respectively.

## Figures and Tables

**Table 1:** Parameters considered in the previous experimental investigations.

Parameter	Source
concrete strength	[8,10,11,12]
Type of aggregate, composition of the aggregate: ALWC (All-lightweight aggregate concrete), SLWC (Sanded-lightweight aggregate concrete), RAC (Recycled aggregate concrete)	[13,14,15,16,17,18]
Interface between ordinary and lightweight aggregate concrete	[3,19]
Shear reinforcement ratio, diameter, and location of the reinforcement	[8,11,14,15,16,20,21,22,23]
Roughness of the interface	[11,14,15,22,24,25,26]
Embedment depth and installation mode of the shear reinforcement	[11,23]
External pressure	[16,24]
Shape of the composite beam	[27,28,29]
Location of the interface within the section of the composite beam	[28,29]

**Table 2:** Coefficients depending on the surface roughness.

Surface Preparation	EN 1992-1-1 [4]		*fib* Model Code 2010 [32]					PrEN 1992-1-1:2020 [33]				
	*μ*	*c*	*μ*	*c_a_*	*c_r_*	*κ*_1_	*κ*_2_	*μ_v_*	*c_v_*_1_	*c_v_*_2_	*k_t_*	*k_f_*
Very rough	0.9	0.5	0.8 (1.0)	0.5	0.2	0.5	0.9	0.9	0.19	0.15	0.5	0.9
Rough	0.7	0.4	0.7	0.4	0.1	0.5	0.9	0.7	0.15	0.075	0.5	0.9
Smooth	0.6	0.2	0.6	0.2	0	0.5	1.1	0.6	0.075	0	0.5	1.1
Very smooth	0.5	0.025 –0.10	0.5	0.025	0	0	1.5	0.5	0.0095	0	0	1.5

**Table 3:** Types of concrete interfaces and corresponding conditions of preparation [32,33].

Roughness	Requirements for Surface Preparation
Very smooth	Surface against, e.g., steel or plastic formwork
Smooth	Surface without treatment after compacting (*R_z_* < 3 mm; *R_t_* < 1.5 mm)
Rough	Surface achieved by raking or exposing aggregate (*R_z_* ≥ 3 mm at maximum 40 mm spacing; *R_t_* ≥ 1.5 mm)
Very rough	Achieved by raking or exposing aggregate (*R_z_* ≥ 6 mm at maximum 40 mm spacing; *R_t_* ≥ 3 mm)

**Table 4:** Coefficients corresponding to various interface conditions according to AASHTO [35].

Type of Connection	Normal-Weight Concrete Placed Monolithically	Cast-in-Place Concrete Slab on Clean Concrete Girder Surfaces	Normal-Weight Concrete Placed Against Surface Intentionally Roughened (*R_z_* > 6.4 mm)	Lightweight Aggreg. Concrete Monolithic or Placed Against Surface Intentionally Roughened (*R_z_* > 6.4 mm)	Concrete Placed Against a Clean Concrete Surface Not Intentionally Roughened (*R_z_* < 6.4 mm)
*c* (MPa)	2.76	1.93	1.65	1.65	0.52
*μ*	1.4	1.0	1.0	1.0	0.6
*K*_1_	0.25	0.3	0.25	0.25	0.2
*v_u,max_* (MPa)	10.34	12.41 ^1^/8.96 ^2^	10.34	6.89	5.52

^1^ Normal-weight concrete (NWC), ^2^ lightweight aggregate concrete (LWAC).

**Table 5:** Experimental program.

Designation of the Specimen	Concrete Overlay	Roughness *R_t_* (mm)	Shear Reinforcement *ρ_s_* (%)
L30/50-S-0	Lightweight Aggregate Concrete LC30/33	<0.1	none
L30/50-S-0 bis			
L30/50-S-0.14			0.14
L30/50-R-0		>0.7	none
L30/50-R-0 bis			
L30/50-R-0.14			0.14
L30/30-S-0	Lightweight Aggregate Concrete LC25/28	<0.1	none
L30/30-S-0 bis			
L30/30-S-0.14			0.14
L30/30-R-0		>0.7	none
L30/30-R-0 bis			
L30/30-R-0.14			0.14

**Table 6:** Recipe of concrete mixes.

Ingredient	Content per 1 m^3^ (kg)		
	NWC (Substrate)	LWAC (Overlay)	
		L30/50 Series	L30/30 Series
**cement**	270 (CEM I 52.5N)	270 (CEM I 52.5N)	280 (CEM I 42.5N)
Water	180	185	190
Sand 0–2 mm	800	605	610
Crushed granite 2–8 mm	1070	–	–
Aggregate “Certyd” 4–9 mm	–	620	650
Zeolite 50	–	15	–
Fly ash	–	70	130
Plasticizer	1.5% c.m.	1.8% c.m.	0.5% c.m.

**Table 7:** Properties of hardened concrete.

Substrate (NWC)			Overlay—L30/50 Series (LWAC)				Overlay—L30/30 Series (LWAC)			
*f_cm_* (MPa)	*f_ctm_* (MPa)	*E_cm_* (GPa)	*f_lcm_* (MPa)	*f_lctm_* (MPa)	*E_lcm_* (GPa)	*ρ* (kg/m^3^)	*f_lcm_* (MPa)	*f_lctm_* (MPa)	*E_lcm_* (GPa)	*ρ* (kg/m^3^)
39.9 (25/2.0%)	3.49 (28/4.2%)	30.0 (23/4.2%)	51.6 (6/2.8%)	4.04 (10/5.2%)	19.8 (3/1.1%)	1787 (4/0.5%)	32.2 (6/4.5%)	2.13 (5/8.5%)	18.2 (3/2.5%)	1659 (3/0.6%)

(Values in parentheses describe: Number of samples and corresponding coefficient of variation characterizing the test results.)

**Table 8:** Load carrying capacities and corresponding deformation characteristics.

Series Designation	L30/50 Series						L30/30 Series					
	Smooth Interface			Rough Interface			Smooth Interface			Rough Interface		
	L30/50-S-0	L30/50-S-0 bis	L30/50-S-0.14	L30/50-R-0	L30/50-R-0 bis	L30/50-R-0.14	L30/30-S-0	L30/30-S-0 bis	L30/30-S-0.14	L30/30-R-0	L30/30-R-0 bis	L30/30-R-0.14
*F_exp_* (kN)	143.0	86.0	154.0	135.0	74.0	87.0	32.5	38.3	50.0	51.5	68.5	50.0
*v_m_* (mm)	0.021	0.012	0.012	0.016	0.018	0.020	0.004	0.004	0.004	0.006	0.012	0.007
*w_m_* (mm)	0.008	0.005	0.011	0.006	0.005	0.007	0.004	0.004	0.005	0.004	0.004	0.008
*F_res_* (kN)			35.1			27.2			32.4			33.4
*v_max_* (mm)			7.268			11.510			11.848			6.973
*w_max_* (mm)			2.188			1.777			2.002			1.696

(*F_exp_*—maximum load carrying capacity, *F_res_*—residual (post-peak) load carrying capacity, *v_m_*, *w_m_*—vertical displacement and crack width at failure or formation of the macrocrack, *v_max_*, *w_max_*—slip and crack width at failure.)

**Table 9:** Steel strain and stress at peak load.

Interface Condition	Smooth		Rough	
Specimen	L30/30-S0.14	L30/50-S0.14	L30/30-R0.14	L30-50-R0.14
*ε_si_* (%)	0.551	0.686	gauge failure	−0.032
*σ_s_* (MPa)	106.8	132.9		−6.2
*σ_s_*/*f_ym_*	0.18	0.23		0.01

## Data Availability

The data presented in this study are available on request from the corresponding author.

## List of Designations

*A_s_*	cross section of the rebar
*A_s,min_*	minimal reinforcement
*c*	factor reflecting adhesion forces
*c_a_, c_v_*_1_	coefficient for adhesive bond
*c_r_, c_v_*_2_	coefficient for aggregate interlock effect
*D*	diameter of the circle formed in sand path method
*D_g_*	maximum size of the aggregate
*E_cm_*	secant modulus of elasticity of ordinary concrete
*E_lcm_*	secant modulus of elasticity of lightweight aggregate concrete
*E_s_*	Young’s modulus of reinforcing steel
*f_cd_*	concrete compressive strength (design value)
*f_cm_*	concrete compressive strength (mean value)
*f_ck_*	concrete compressive strength (characteristic value)
*f_ctd_*	tensile strength of ordinary concrete (design value)
*f_ctm_*	tensile strength of ordinary concrete (mean value)
*f_ct,spm_*	tensile splitting strength of concrete (mean value)
*f_lcm_*	compressive strength of lightweight aggregate concrete (mean value)
*f_lctm_*	tensile strength of lightweight aggregate concrete (mean value)
*f_yd_*	yield strength of the reinforcement (design value)
*f_yk_*	yield strength of the reinforcement (characteristic value)
*f_ym_*	yield strength of the reinforcement (mean value)
*f_um_*	tensile strength of the reinforcement (mean value)
*F*	force applied on the test specimen
*F_c_*	load capacity resulting from concrete contribution
*F_calc_*	theoretical load carrying capacity (resulting from design procedure)
*F_exp_*	maximum load carrying capacity
*F_res_*	residual (post-peak) load carrying capacity
*F_s_*	load capacity resulting from steel contribution
*h_ef_*	embedment depth of the shear reinforcement
*k_f_*	coefficient for dowel action
*k_t_*	coefficient for clamping action
*K*_1_	factor reflecting fraction of concrete strength available to resist interface shear
*R_pm_*	mean peak height
*R_t_*	mean profile depth
*R_z_*	mean peak-to-valley height
*t_min_*	smaller value of the thickness of new and old concrete layer
*w_m_*	crack width
*w_max_*	crack width at failure
*v*	coefficient for the strength reduction of the compressed struts
*v_m_*	slip at the interface
*v_max_*	slip at failure
*v_Rd,i_*	shear resistance of the interface
*v_u_*	interface shear resistance
*v_u,max_*	limiting interface shear resistance
*V*	volume of sand used in sand path method
*α*	inclination of the reinforcement to the shear plane
*ε_si_*	strain of shear reinforcement
*ε_yh_*	steel strain at the end of yield plateau
*ε_ym_*	steel strain at yield point
*ε_um_*	steel strain at peak stress
*γ_c_*	partial safety factor for concrete
*κ*_1_	interaction coefficient
*κ*_2_	coefficient for flexural resistance
*λ*	coefficient reflecting aggregate composition
*μ, μ_v_*	coefficient of friction
*ρ*	concrete density (oven dry)
*ρ_s_*	ratio of the reinforcement crossing shear plane
*ρ_s,min_*	minimal shear reinforcement ratio
*σ_n_*	stress acting perpendicular to the shear plane
*σ_s_*	steel stress
*τ*	shear stress
*τ_Rd,i_*	shear resistance of the interface
*τ_Rd,max_*	maximum shear resistance of the interface
